# Visual Impairment Due to Bilateral Multifocal Choroidal Metastasis of Parotid Adenocarcinoma: A Case Report

**DOI:** 10.3389/fonc.2014.00136

**Published:** 2014-06-05

**Authors:** Gerard Walls, Seamus Napier, David Stewart

**Affiliations:** ^1^Northern Ireland Cancer Centre, Belfast, UK; ^2^Department of Histopathology, Royal Group of Hospitals Trust, Belfast, UK

**Keywords:** parotid neoplasms, external beam radiotherapy, choroid neoplasms, radiation effects, orbital neoplasms

## Abstract

**Background:** Orbital metastases are an uncommon finding, being present in just 9% deceased patients with metastatic cancer. Only a quarter of patients with choroidal metastases have bilateral disease. Parotid cancer is not a common form of head and neck malignancy. Bilateral multifocal metastases from adenocarcinoma ex pleomorphic of parotid gland have been documented just once before in the literature. We present a similar case where palliative EBRT was used to gain local control with minimal toxicity.

**Case Presentation:** The case of a 45-year-old Caucasian gentleman who presented to his general practitioner with otalgia and weight loss. Imaging revealed a mass in the deep lobe of the left parotid gland, invading into the medial pterygoid muscle. PET–CT revealed locoregional and distant lymphatic involvement plus disseminated skeletal metastases. Lymph node examination revealed adenocarcinoma ex pleomorphic histology. Within weeks of this diagnosis, the patient developed rapidly progressive visual impairment. Ophthalmologists found multifocal uveal masses bilaterally. Palliative fractions of external beam radiotherapy were delivered to the orbits before combination chemotherapy. We discuss the patient’s presentation, histopathology, and management, with support from the literature regarding applied and related therapies.

**Summary:** In this rare presentation of disseminated malignancy affecting the choroid bilaterally, the authors demonstrate the application of palliative EBRT to good local effect. Given the nature of this treatment and that of the metastases, in the setting of incurable disease orbital morbidity is likely to occur again before the patient’s death. Awareness of the initial symptoms is important to provide prompt care and maintain quality of life.

## Introduction

Macroscopic orbital metastases are an uncommon clinical finding, but an autopsy series of patients with metastatic disease have found microscopic metastases in approximately 9% of cases (1). The uvea, the vascular middle layer of the eye is the most common site for orbital metastases. In a survey of 520 eyes (2) with uveal metastases, the choroid was involved in 88%, the iris in 9% and the ciliary body in 2%. Tumors most often occurred in the posterior pole of the eye with an average of two tumors per eye. A third of patients found to have orbital metastases have not yet had a previous diagnosis of cancer (1). Only a quarter of patients with choroidal metastases have bilateral disease (3), and these are most likely to come from breast and lung primary malignancies. In 42% of patients diagnosed with orbital metastases, there was no previous evidence of systemic disease (4). After an established diagnosis of orbital disease, there was a mean survival of 15 months. Parotid malignancy of acinic cell, mixed type, adenoid cystic, epidermoid, and adenocarcinoma histologies affecting the uvea have been presented as cases in the literature, all as monocular metastases. Bilateral choroidal metastases in adenocarcinoma of the parotid gland have been reported just once before to the best knowledge of the authors, 1 year following resection and irradiation of the patient’s tumor (5). Treatment in the case reports includes surgical removal, enucleation, exenteration, plaque radiotherapy, and conservative management. Herein, we describe the use of palliative external beam radiotherapy to the orbits for bilateral painless loss of vision caused by uveal metastases of parotid malignancy in a young patient.

## Case Presentation

**Figure 1:**
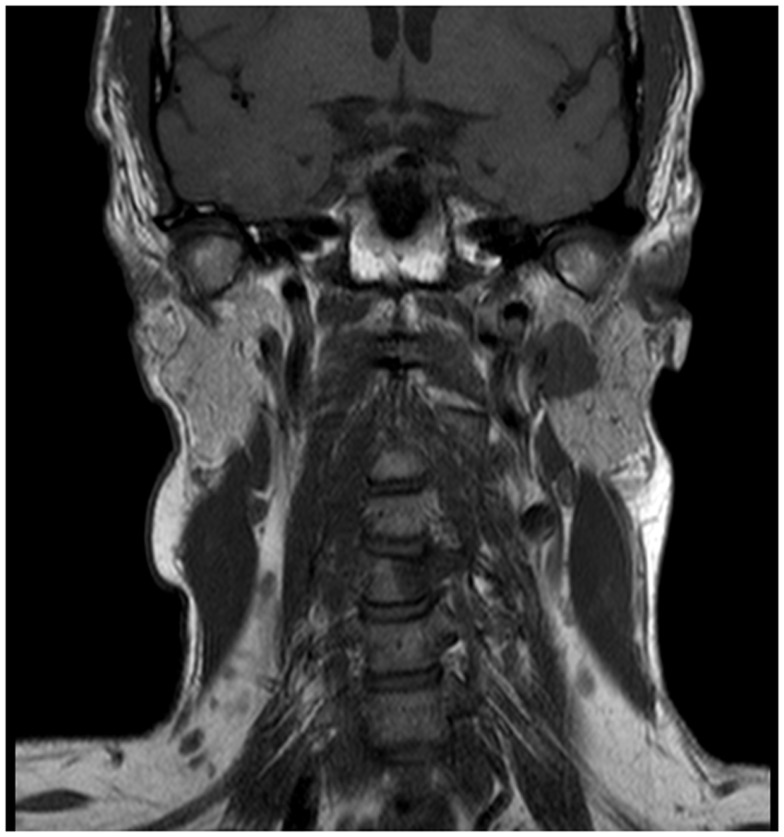
**Magnetic resonance imaging showing the left parotid gland mass in the coronal plane**.

**Figure 2:**
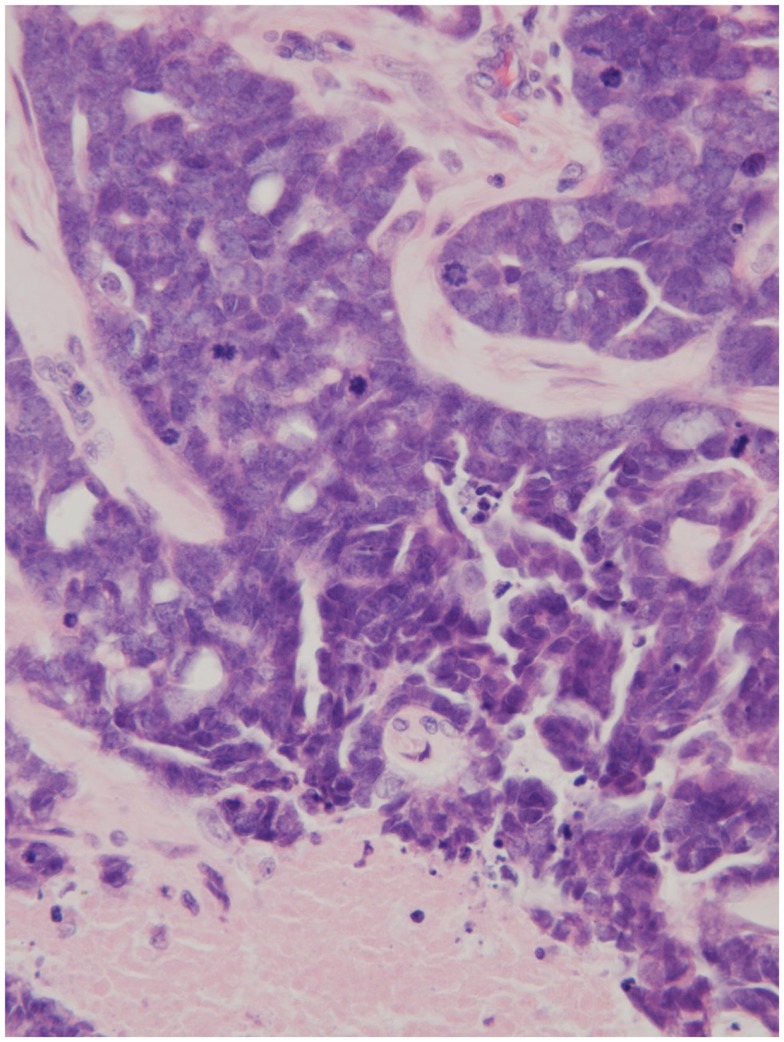
**Node histology (×250) demonstrating necrotic tissue, an abundance of mitotic figures, and the impression of glandular architecture (H&E)**.

**Figure 3:**
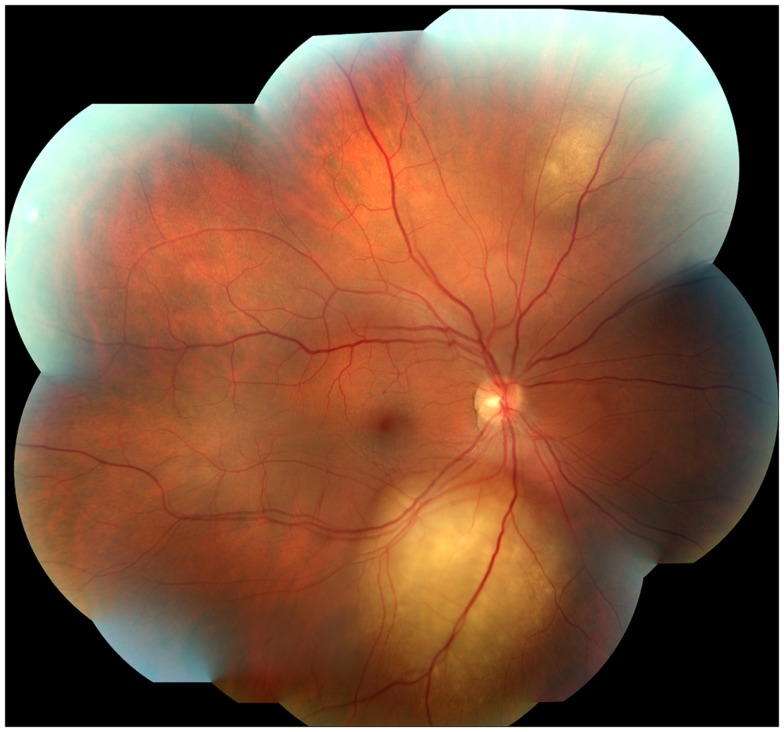
**Photograph of the right fundus, with evidence of two uveal metastases**.

A 45-year-old male architect and non-smoker presented to his General Practitioner with a history of intermittent otalgia, and weight loss of 20 kg in 1 year, attributed to wisdom tooth eruption and lifestyle changes, respectively. He denied all other ear, nose, and throat symptoms. Physical examination, including cranial nerves, was unremarkable and routine blood parameters were within normal limits. Assessment by an ENT surgeon was also unremarkable but contrast magnetic resonance imaging of the head revealed a 22 mm × 13 mm lesion in the deep lobe of the left parotid gland invading into the medial pterygoid muscle and causing effacement of the left parapharangeal space (see Figure 1). Fine needle aspiration of the salivary gland lesion was suggestive of adenoid cystic carcinoma and referral to clinical oncology was made. An FDG PET–CT study was performed and showed an FDG-avid soft tissue lesion in deep lobe of left parotid of dimensions 2.0 cm × 2.5 cm with a SUVmax of 8.3. There was an impression of internal calcification suggesting a longstanding lesion. There was left sided cervical lymphadenopathy, thoracic lymphadenopathy, and disseminated skeletal metastases. Biopsy of two cervical nodes showed high grade metastatic adenocarcinoma likely from a carcinoma ex pleomorphic adenoma (see Figure 2). During staging investigations, the patient presented to an acute eye service complaining of bilateral, markedly reduced visual acuity without pain. His previously 6/6 vision had deteriorated to 6/15 (right) and 6/60 (left) [note that 6 m are used in UK in contrast to 20 feet in the USA]. On fundoscopy, exudative retinal detachment was observed bilaterally (see Figure 3). Fluorescein angiography identified the cause as multiple choroidal metastases bilaterally. In the left eye, the fovea was involved.

## Treatment and Outcomes

Due to the diffuse nature of the orbital metastases, palliative EBRT was offered. The patient received 20 Gray of external beam radiotherapy to his orbits in five fractions, using 6 MV photons in parallel opposed lateral fields. Following administration of orbital irradiation, the patient’s visual acuity exhibited recovery to 6/9 on the left and 6/5 on the right, both retinas became completely attached and the choroidal masses were flattened. The only side effect experienced was dry eyes, likely due to lacrimal gland irradiation. The patient was of performance status 1 and went on to receive six cycles of epirubicin, cisplatin, and 5-fluorouracil chemotherapy plus palliative radiotherapy to bone metastases when needed. He has recently completed his chemotherapy. The patient remained at performance status 1 throughout his treatment and experienced no significant side effects from treatment, allowing him to continue to work part-time. CT imaging after cycle 6 showed stable disease and at 6 months post-diagnosis his vision remained stable.

## Discussion

Tumors of the salivary glands account for approximately 5% of all head and neck neoplasms. The average age of patients with malignant neoplasm is approximately 55 years of age; for benign tumors, approximately 40 years. One-quarter of parotid neoplasms are malignant. Metastases are present in 20–25% of patients at the time of diagnosis. Painless loss of visual acuity is the presenting feature of uveal metastases in 75% patients with primary disease of the breast (6). No symptoms are experienced in 7% cases. The most common primary sites likely to contribute metastases to the orbit can be viewed in Table 1 (2).

**Table 1:** **The most common sources of metastases to the choroid**.

Primary site	Number of men	Number of women	Total (%)
Breast	2	194	196 (47)
Lung	55	35	90 (22)
Gastrointestinal	13	5	18 (4)
Kidney	8	1	9 (2)
Skin (melanoma)	5	4	9 (2)
Prostate	9	0	9 (2)
Others	5	11	16 (4)
Unknown	40	33	73 (17)
Total	137	283	420

Common sites of metastasis for carcinoma ex pleomorphic include lung, bone, and liver (7). Choroidal metastases in this patient may be considered evidence of hematogenous dissemination further to the widespread bone disease, as the orbit has a limited lymphatic system (8). The areas of calcification observed in some tissue samples and on PET–CT indicate that the patient most likely had an undetected pleomorphic adenoma for some time. These tumors’ benign nature and indolent course with potential for malignant transformation are well-known. These tumors typically present later than seen in this case, in the sixth to eighth decades.

Orbital irradiation is the main treatment option for choroidal metastases and is mostly delivered by external beam irradiation. Stereotactic radiation therapy is also an option but would be difficult to deliver in the emergency situation when patients present with acute visual loss and also on account that metastases are usually multiple and also bilateral in half of cases. Stereotactic radiotherapy is also associated with significantly higher rates of orbital morbidity at 4 years, in a study of orbital melanoma (9). Response rates to orbital radiation treatment are high but overall survival is generally poor on account of systemic disease. A retrospective case series of 264 breast cancer patients with uveal metastases showed regression in 64% cases following external beam radiotherapy, and 65% following systemic treatments (6). External beam radiotherapy technical factors such as higher biologically effective dose, lower treatment energy, and whole globe (versus lens-sparing) technique do not predispose eyes to complications of irradiation including cataracts (10). This is likely to be related to the poor survival rates for malignant disease with choroidal secondaries.

Irradiation is also administered by brachytherapy, using surgically introduced (and removed) plaques (2).This data delineates effectiveness both as primary treatment, and as salvage treatment when EBRT has failed. Radiation retinopathy, radiation papillopathy, or both were found in three patients (8%) and occurred at a mean of 8 months after treatment. Similar results were found in a more recent study quantifying EBRT toxicity (11). Other authors indicate that the development of pathology in keeping with radiation retinopathy is related to tumor volume (12), based on findings of up to 60% patients receiving plaque therapy developing the complication.

A group at the Liverpool Ocular Oncology Centre enjoyed success in two patients using Ruthenium-106 included in a recently published study (13). It was in this center over two decades ago that proton therapy was first used as a treatment of malignancy. Data from the Loma Linda centre in California indicates that proton therapy can be used safely and effectively to treat uveal primary tumors (14). In a study of 78 singular orbital melanomas, local control at 5 years was found by authors there to be 90.5%, and eye preservation was achieved in 75.3%. Useful visual acuity remained with 49.1% at the 5-year point.

Other centers use laser therapies in the form of transpupillary thermotherapy, photocoagulation, and photodynamic therapy (15), however, data on these limited at present. As cancer survivorship continues to improve and the incidence of orbital metastases increases, these treatments may play an important part in the future management of orbital metastases.

Unfortunately, in some clinical settings enucleation or exenteration is the most appropriate management. Due to the diffuse nature of the metastases in the case presented, it was felt EBRT offered the best chance of local control. Combination chemotherapy was offered to gain control of systemic disease – with the aim of extending survival. Throughout this time period, the clinicians involved must be vigilant for orbital morbidity, as a result of local relapse, and of the EBRT outlined (16, 17).

There are very few clinical trials investigating the use of systemic therapy in salivary gland cancers. Combination chemotherapy with epirubicin, cisplatin, and 5-fluorouracil is a regime used in the Northern Ireland Cancer Centre for metastatic salivary gland carcinoma and objective responses seen are similar to the literature at approximately 30% but are of short duration. Objective response rates are similar to other regimens reported in the literature. Demirci et al. (6) found systemic survival rates for breast cancer patients with uveal metastasis were 65% at 1-year, 34% at 3-year, and 24% at 5-year follow-up.

## Concluding Remark

In summary, the above case demonstrates that new visual signs or symptoms should be investigated thoroughly in patients with parotid malignancy and good responses to orbital irradiation can be obtained with palliative fractionation regime.

## Conflict of Interest Statement

The authors declare that the research was conducted in the absence of any commercial or financial relationships that could be construed as a potential conflict of interest.
